# Food insecurity during COVID-19 pandemic: A genuine concern for people from disadvantaged community and low-income families in Province 2 of Nepal

**DOI:** 10.1371/journal.pone.0254954

**Published:** 2021-07-21

**Authors:** Devendra Raj Singh, Dev Ram Sunuwar, Sunil Kumar Shah, Lalita Kumari Sah, Kshitij Karki, Rajeeb Kumar Sah

**Affiliations:** 1 Department of Public Health, Asian College for Advance Studies, Purbanchal University, Lalitpur, Nepal; 2 Research and Innovation Section, Southeast Asia Development Actions Network (SADAN), Lalitpur, Nepal; 3 Research Section, Swadesh Development Foundation (SDF), Siraha, Province-2, Nepal; 4 Department of Nutrition and Dietetics, Armed Police Force Hospital, Kathmandu, Nepal; 5 Program Section, Bagmati Welfare Society Nepal, Sarlahi, Province-2, Nepal; 6 Faculty of Medicine, Health and Social Care, Canterbury Christ Church University, Canterbury, Kent, United Kingdom; 7 Department of Allied Health Professions, Sports and Exercise, School of Human and Health Sciences, University of Huddersfield, Huddersfield, United Kingdom; Shahjalal University of Science and Technology, BANGLADESH

## Abstract

**Background:**

Food insecurity is a serious social and public health problem which is exacerbated by the COVID-19 pandemic especially in resource-poor countries such as Nepal. However, there is a paucity of evidence at local levels. This study aims to explore food insecurity among people from the disadvantaged community and low-income families during the COVID-19 pandemic in Province-2 of Nepal.

**Methods:**

The semi-structured qualitative interviews were conducted virtually among purposively selected participants (n = 41) from both urban and rural areas in eight districts of Province 2 in Nepal. All the interviews were conducted in the local language between July and August 2020. The data analysis was performed using thematic network analysis in Nvivo 12 Pro software.

**Results:**

The results of this study are grouped into four global themes: i) Impact of COVID-19 on food security; ii) Food insecurity and coping strategies during the COVID-19 pandemic, iii) Food relief and emergency support during the COVID-19 pandemic, and iv) Impact of COVID-19 and food insecurity on health and wellbeing. Most participants in the study expressed that families from low socioeconomic backgrounds and disadvantaged communities such as those working on daily wages and who rely on remittance had experienced increased food insecurity during the COVID-19 pandemic. Participants used different forms of coping strategies to meet their food requirements during the pandemic. Community members experienced favouritism, nepotism, and partiality from local politicians and authorities during the distribution of food relief. The food insecurity among low-income and disadvantaged families has affected their health and wellbeing making them increasingly vulnerable to the COVID-19 infection.

**Conclusion:**

Food insecurity among low-income and disadvantaged families was found to be a serious problem during the COVID-19 pandemic. The study suggests that the relief support plan and policies should be focused on the implementation of immediate sustainable food security strategies to prevent hunger, malnutrition, and mental health problems among the most vulnerable groups in the community.

## Background

The COVID-19 pandemic has aggravated the situation of food insecurity resulting in adverse public health consequences globally [1–3]. Food insecurity refers to a lack of consistent access to enough food for an individual or family due to limited financial or other resources [4]. The United Nations Food Agriculture Organization (FAO) suggests food insecurity is a multifaceted phenomenon that occurs due to the disruptions in availability, access, utilization, and stability of food [5]. The World Food Program (WFP) estimated that about 272 million people are trapped in acute food insecurity and 97 million people suffered from chronic food insecurity across the globe in 2020 directly due to the aggravating impact of the COVID-19 pandemic [6]. Food insecurity and hunger have several potential adverse health and social consequences such as the risk of malnutrition, multiple infections, chronic diseases, poor health and wellbeing, impaired learning, poor mental health, conflicts in society, increased social and economic inequalities, and has the potential to hinder the overall developmental activities among children and young people [7, 8]. This explicitly indicates that timely contextual evidence-based effective interventions are required to minimize the impact of COVID-19 on food security that would support to meet the Global Nutrition Targets 2025 and Sustainable Development Goal (SDG) 2: Zero hunger [9, 10].

Nepal is among the least developed countries (LDC) and lies in the lap of the Himalayas between the two huge economies, India and China. In the past decades, Nepal has suffered from extreme poverty, food insecurity, and malnutrition caused by man-made and natural disasters such as political instability and earthquakes [11]. The COVID-19 pandemic has exacerbated the food insecurity situation in different parts of Nepal. As of June 10, 2021, it is reported that there are 598,813 diagnosed COVID-19 cases with 8,179 deaths due to COVID-19 in Nepal where total population of country is 29,622,327 [12]. Nepal went into complete lockdown at the end of March 2020, which lasted for 120 days, followed by a partial lockdown until September 2020 [13]. The complex situation prompted by COVID-19 lockdown measures for the containment of the pandemic has produced a global economic downturn, disruption of supply chains, interruption of social protection schemes, uneven food prices, changes in productivity, altered food environments, and increased economic inequalities that have posed serious threat to meet the daily food and nutrition requirements for most vulnerable population across the world, including Nepal [2, 14, 15]. In addition, the situation of the food crisis has been exacerbated further among families who were already facing food insecurity before the pandemic [6]. Nepal government’s recent report showed that three out of ten households lost their income activity and about 58% of the households did not have food stocks for more than one month during the COVID-19 crisis [16].

The Province- 2 of Nepal where this study was conducted has one of the highest percentages (≈ 30%) of household who have their family member migrated for labour work in a foreign land such as India and Gulf countries like Malaysia, United Arab Emirates, Qatar, Saudi Arabia, Bahrain, and other countries [17]. Nepal was the19^th^ largest remittance receiver country in the world in 2018 [18]. Nepal’s economy in the recent decades has become remittance dependent contributing to about 25% of the national economy [19]. The most recent report (2020) by the International Labour Organization (ILO) depicts that about 500,000 migrant workers were likely to return home country due to the COVID-19 pandemic [20]. Also, the World Bank warned that the global remittance an income source in developing countries is projected to immediately reduce by 20% in 2020 due to the COVID-19 crisis [21]. Although the remittance flow to Nepal surprisingly increased by 23% despite COVID-19, the numbers of migrant workers returning to Nepal during the pandemic have increased significantly [22, 23] However, there are no adequate plans for income-generating activities for such migrants’ returnees in Nepal [20]. Thus, rising unemployment and increased food prices due to the COVID-19 pandemic are expected to have a huge adverse impact on food security particularly among low-income and disadvantaged Nepalese households where a significant amount of the income is spent on daily food requirements for the family [20]. Although the national COVID-19 lockdown measures have been relaxed, movement of migrant workers and economic activities remains limited, worsening the food security, particularly among low-income and disadvantaged households across the nation [24], and therefore the current health and nutritional impacts of the pandemic are likely to continue beyond the pandemic [3].

Nepal’s poor resilience of commercial agriculture, as well as traditional farming due to high dependency for seeds and fertilizers on the neighbouring countries, has reduced its agricultural productivity posing an increased risk of food insecurity in both rural and urban settings [24]. Although Province 2 is part of the Terai region, also known as the “grain house of the nation”, 48% of its population lives in multidimensional poverty making it the second poorest province of the country [25]. Despite the federal, provincial, and local government’s efforts in mitigating the impacts of the COVID-19 pandemic, the current crisis has severely affected food security among the Nepalese population [16]. However, there is a paucity of local evidence that can elucidate how the COVID-19 has impacted food security and people’s wellbeing in Province 2 of Nepal. To the best of our knowledge, this is the first qualitative study that aims to explore food insecurity among people from the disadvantaged community and low-income families during the COVID-19 pandemic in Province-2 of Nepal. A social-ecological framework [26], which captures five levels of factors: intrapersonal factors, interpersonal factors, organization, community and policy level factors, is used in this paper to analyze the complex phenomenon responsible for food insecurity among the low-income and disadvantaged families during the COVID-19 pandemic [27].

## Methods

The study used qualitative research to explore food insecurity during the COVID-19 pandemic, particularly among people from low-income and disadvantaged families in Province-2 of Nepal. The study followed the guidelines for standard Consolidated Criteria for Reporting Qualitative Studies (COREQ) [28]. The ethical approval for this study was obtained from the ethical review board of the Nepal Health Research Council (Ref. no: 2658). Both written and verbal consent was obtained from each participants.

### Study setting

**Fig 1:**
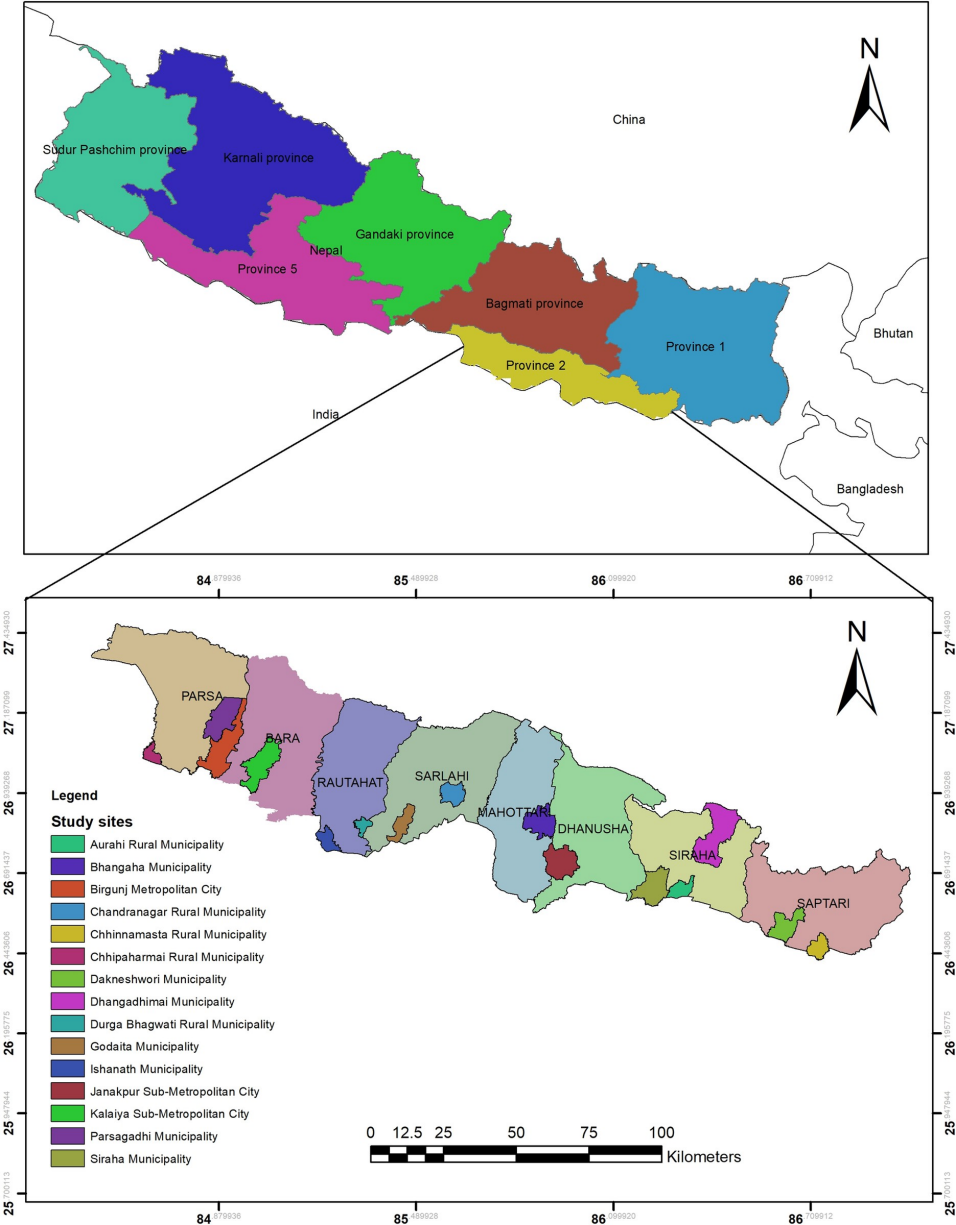
Map showing urban and rural study sites in eight districts in Province 2 of Nepal. The map was created using ArcGIS software V.10.8, and base files of the administrative provinces and districts of Nepal were obtained from the Government of Nepal, Ministry of Land Management, and Natural Earth, that is publicly available without restrictions (http://dos.gov.np/nepal-map) [35].

This study was conducted in both rural and urban sites across all eight districts of Province 2, located in the Southern part of Nepal (also known as the Terai region) (Fig 1). It has a total population of 5,404,145, covers an area of 9,661 km^2^, and has an open Southern border with India which has high movements of migrant workers [29]. The majority of the inhabitants in the province belong to Madheshi ethnic groups and speak Maithili, Bhojpuri, Bajjika, Nepali, Abhadhi, and other local languages [30]. The eight districts of the Province-2 are further divided into self-governed one metropolitan city, three sub-metropolitan cities, 73 municipalities, and 59 rural municipalities [30]. The most recent Nepal Demographic and Health Survey 2016 showed, about 57% of households in Province 2 had experienced some forms of food insecurity which is relatively higher than the national figure (52%) [31]. Also, the province lies at the bottom of the list for most health indicators and has poor children and adult nutritional status compared to other regions of the country [31]. In addition, the province has a low literacy rate of 56% and more than half of the province population lives in multidimensional poverty [25, 32]. Moreover, the province had nearly 50% of the total COVID-19 cases of Nepal during the period of April-August 2020 [33]. The high proportion of COVID-19 cases in Province-2 was mainly due to the share of porous border with India at all eight districts and significant number of labour migrant returnees [34].

### Research participants

A total of 41 participants were purposively [36] selected from all eight districts of Province 2. The purposive sampling in this study provided an opportunity to include diverse participants in terms of sex, age, education, occupation, and residence (Table 1).

**Table 1:** Characteristics of study participants.

Participant No. (P#)	Sex	Age	Education	Occupation	Rural/Urban Municipality	District
P 1	F	47	Secondary level	FCHV	Urban	Saptari
P 2	F	21	Higher secondary level	Student	Urban	Saptari
P 3	M	40	Secondary level	Healthcare worker	Urban	Saptari
P 4	M	48	Higher secondary level	Ward Chairperson	Urban	Saptari
P 5	M	42	Secondary level	Health Coordinator	Rural	Saptari
P 6	F	25	Higher secondary level	Unemployed	Rural	Saptari
P 7	M	28	Higher secondary level	Social Mobiliser	Rural	Saptari
P 8	F	43	Secondary level	FCHV	Rural	Saptari
P 9	M	38	Secondary level	Ward Chairperson	Rural	Saptari
P 10	F	24	Higher secondary level	Healthcare worker	Urban	Parsa
P 11	F	45	No formal education	Farmer	Urban	Parsa
P 12	F	25	No formal education	Housewife	Urban	Parsa
P 13	M	34	University education	Teacher	Urban	Siraha
P 14	F	45	No formal education	Wage based labor	Urban	Siraha
P 15	F	40	Secondary level	FCHV	Rural	Sarlahi
P 16	F	24	Secondary level	Student	Rural	Sarlahi
P 17	M	71	Primary level	Ward Chairperson	Rural	Sarlahi
P 18	M	32	Higher secondary level	Farmer	Rural	Sarlahi
P 19	M	40	University education	Farmer	Urban	Sarlahi
P 20	M	62	Primary level	Ward Chairperson	Urban	Sarlahi
P 21	M	32	Primary level	Tailoring	Urban	Sarlahi
P 22	F	32	No formal education	Farmer	Urban	Sarlahi
P 23	F	40	Primary level	FCHV	Urban	Sarlahi
P 24	M	47	Secondary level	Social Worker	Urban	Parsa
P 25	F	45	University education	Social Worker	Urban	Parsa
P 26	F	30	University education	Journalist	Urban	Dhanusha
P 27	F	40	University education	Social Worker	Urban	Parsa
P 28	M	61	University education	Retired Civil Worker	Urban	Dhanusha
P 29	M	55	Secondary level	Farmer	Rural	Siraha
P 30	M	37	University education	Ward Chairperson	Urban	Siraha
P 31	M	37	University education	Teacher	Urban	Siraha
P 32	M	40	University education	Farmer	Rural	Siraha
P 33	M	47	Higher secondary level	Farmer	Rural	Rautahat
P 34	M	27	Higher secondary level	Health worker	Urban	Rautahat
P 35	F	38	Secondary level	Housewife	Rural	Rautahat
P 36	M	51	University education	Teacher	Urban	Bara
P 37	F	36	University education	Human right Activist	Urban	Bara
P 38	M	27	Higher secondary level	Health Worker	Urban	Mohattari
P 39	M	50	Higher secondary level	Farmer	Urban	Mohattari
P 40	F	29	University education	Health worker	Urban	Bara
P 41	F	25	Secondary level	FCHV	Urban	Siraha

No formal education: Never attended school, Primary level: Grade (1–5), Secondary level: Grade (6–10), Higher secondary level: Grade (11–12 or equivalent), University education: Undergraduate and Postgraduate; FCHV: Female Community Health Volunteer.

### Data collection

Semi-structured qualitative interviews were conducted to collect the data for this study. The semi-structured interview guide allowed flexibility to probe for detailed discussion by remaining focused on the original research topic [37]. All interviews were conducted between July and August 2020. To avoid the risk of COVID-19 transmission during the face-to-face interviews, a virtual communication tool such as a mobile phone was used to conduct all interviews [38]. Participants’ contact numbers were obtained from the local ward office. The interviewers made a phone call to participants first for obtaining verbal consent and appointments, and later second phone call so that participants feel comfortable for the interview. Each virtual interview lasted for about 25–30 minutes. The interviews were conducted in local languages such as Awadhi, Bhojpuri, Maithili, and Nepali languages using pretested interview guidelines by the authors DRSi (MSc, MA), SKS (MPH), and DRSu (MSc) with the supervision from RKS (MSc, PhD), who are fluent in the local languages. Each interview were recorded on mobile phone and the files were transferred to the password-protected folder on the computer. Each interview was transcribed and translated into the English language for analysis purposes. The quality and completeness of the transcription and translation of the interviews were cross-checked independently by RKS. This study is second part of the previous study that aimed to explore community perceptions of COVID-19 and their experiences towards health services utilization during the pandemic in Province-2 of Nepal [39].

### Data analysis

The data were analysed using thematic network analysis [40] in NVivo 12 Pro (QRS International, London, United Kingdom). The data analysis started after the completion of 25^th^ interview, where an inductive process was used to generate themes. The first seven interviews were coded independently by DRSi, LKS, and RKS to identify basics themes and avoid lone researcher bias in qualitative research [41]. Data saturation was reached after the 38^th^ interview, which meant no new basic themes were identified after that [42]. The basic themes indicating similar issues were clustered together to create organizing themes, which enhanced the meaning and significance of the organized texts. Finally, the global themes were generated from organizing themes by using thematic network analysis [40]. The interpretation and arguments on specific issues were presented under four separate global themes (S1 Table).

## Results

Using thematic network analysis [40] the findings from this qualitative research are presented under four global themes: i) Impact of COVID-19 on food security; ii) Food insecurity and coping strategies during the COVID-19 pandemic, iii) Food relief and emergency support during the COVID-19 pandemic, and iv) Impact of COVID-19 and food insecurity on Health and Wellbeing.

### Impact of COVID-19 on food security

Most participants in this research highlighted that families from low socioeconomic backgrounds such as those working on daily wages were the most affected population groups due to the loss of their jobs, as all local businesses and markets were closed during the lockdown due to the COVID-19 pandemic. One of the social workers reported.

*Those who have money*, *they are not affected much and have not experienced hardship but for poor people*, *it is a curse on them*. *Construction sites are closed*, *factories are totally closed and there are no other works available*. *Daily wages labourers from the poorer families are the most affected*.

                 *(Participant 25*, *45 years*, *Female*, *Parsa district)*

Dalits, an ethnically disadvantaged group in Nepal, mostly rely on daily wages and are the poorest in the community. A participant from the Dalit community who was a daily wage worker expressed his experience as,

*Initially*, *people thought this crisis would only last for few weeks but later it extended for months and months and people started facing a food crisis*‥ *we (Dalits) were most affected by this*, *as we lost our job*‥ *there was no income… normally we have 5–6 members in our family to feed*‥ *so there was a problem of food scarcity in our families*. *We were poor even before the COVID-19 crisis and our situation has become more critical after the lockdown*. *In my village*, *most of the families have their family members in India or Gulf countries but we are not receiving any money from them during this crisis period*‥ *we have to borrow money or take a loan from landowners and rich people in our community to survive this crisis…*

                 *(Participant 14*, *45 years*, *Male*, *Siraha district)*

The reliance on remittance to provide a livelihood for these families have been complicated by the COVID-19 crisis, as migrant workers are forced to return home due to the lockdown and lack of job opportunities in their host countries. A journalist describes this situation as widespread and challenging for these migrant workers and their families.

*People don’t have enough employment opportunities here (in Nepal)*. *At least one family member (especially males/husband) from the majority of households in our area has migrated to India or Gulf countries for employment opportunities*. *They are mostly employed in unskilled or labor-based work*, *which has been affected by COVID*. *Many of these migrant workers have returned home or are not sending any money now*.

                 *(Participant 26*, *30 years*, *Female*, *Dhanusha district)*

Food insecurity among families from low socioeconomic conditions was mainly attributed to the financial constraints and also due to the increase in food prices caused by the lockdown. A healthcare worker described this as:

*There were three major reasons for food scarcity in our community*, *first due to loss of jobs among daily wages-based labourers*, *second food prices were suddenly increased to double and triple and thirdly*, *those families who depend on remittance money*, *could not receive remittance on time*.

                 *(Participant 10*, *24 years*, *Female*, *Parsa district)*

Besides the loss of remittance and financial constraints, most participants in this research cited that the increase in food prices were one of the main reasons leading to food insecurity amongst families from low socioeconomic conditions in the community, especially due to limited affordability of food.

*The sudden hike in food prices has led to food shortages for example*: *before lockdown 1 liter of oil cost NRS*. *110 but after lockdown*, *the price rose up to 150 per liter*. *Shopkeepers used to say that they cannot do anything all prices have increased in big markets and therefore we have to increase the prices*. *Before lockdown it was easy to buy foods*, *even people use to go to the border region (India) to buy food-stuffs at cheaper prices but that is not possible now due to lockdown*.

                 *(Participant 3*, *40 years*, *Male*, *Saptari district)*

Other participants highlighted that there were additional factors that have contributed to food insecurity such as border closure, transportation disruption, and lack of food stock.

*An increase in food prices*, *lack of community support*, *transportation disruption*, *border closures*, *lack of food stock at the local market were some of the major reasons for food scarcity in our community*. *Many farmers had to destroy their food stock at the farm because their food stocks such as vegetables*, *fish*, *maize didn’t reach to the market due to lack of transportation or they were compelled to sell these food products at lower prices*. *Farmers sold it because they needed money while others benefitted from it by black-marketing or selling it at higher prices*.

                 *(Participant 18*, *32 years*, *Male*, *Sarlahi district)*

The increase in food prices have resulted in decreased accessibility of food for families from low socioeconomic conditions and disadvantaged community.

*Even if food items were available in the market*, *food prices were increased by 4 times*, *and we could not afford it*. *I have two children and I need to earn money each day to provide food to my family otherwise we have to sleep without food*. *I have no alternative source of income*. *In our community*, *we have 30–40 households that are severely affected by this*. *It is a really very terrible situation for poor families like ours*.

                 *(Participant 22*, *32 years*, *Female*, *Sarlahi district)*

### Food insecurity and coping strategies during the COVID-19 pandemic

Most participants in the study reported to have experienced some forms of food insecurity and they used different coping strategies to meet their food requirements. Those who were well off or has farming lands used their existing food stock during the lockdown.

*As many families in our community depend on agriculture… those who have their own farming lands have food items such as rice*, *pulses*, *and grains in the stock but green vegetables*, *cooking gas was not available due to lockdown*. *Some families have stored some of these food items for a few weeks*, *but this lockdown has now continued for several months*. *This has created food scarcity in even rich and middle-class families*.

                 *(Participant 7*, *28 years*, *Male*, *Saptari district)*

Other participants, especially from disadvantaged and low-income families, relied on the natural environment around them to support with edibles.

*Mushar (Dalit) community started fishing at riverbanks and sell fish at the market during evening time and doing this they earn money to manage food*. *Also*, *they do fishing to manage their food for the family*.

                 *(Participant 24*, *47 years*, *Male*, *Parsa district)*

However, relying on the natural environment was not always enough and therefore families from low socioeconomic conditions, who were the most affected groups due to the pandemic, relied on borrowing money from the landlords and the rich people in the community to secure foodstuff for their families.

*I and my husband have been working on an agricultural farm for my landlord for the last four decades as Haruwah (a kind of bonded labour)*. *We work for our landlord throughout the year and many poor families like ours rely on the landlord even in a normal time*. *During this lockdown*, *my landlord has provided us the necessary amount of rice*, *pulse*, *other foodstuffs*, *and some money for our family*.

                 *(Participant 6*, *25 years*, *Female*, *Saptari district)*

As the pandemic continues and lockdown is extended affecting the daily wage workers for a longer period of time, borrowing from landlords is becoming difficult due to the higher borrowing costs.

*Sometimes rich families provide foodstuffs or monetary support*. *But it’s been so long*, *they all have also taken a loan several times from their landlords in between the lockdown period but now it’s even difficult to ask for money time and again*.

                 *(Participant 35*, *38 years*, *Female*, *Rautahat district)*

Many participants shared their experiences of rationing foods or cutting on meals because they did not have enough resources or money due to the job losses to arrange meals for their families.

*My husband used to work in a jewelry shop on a daily wage but now the shop is closed due to the lockdown… we have no income… initially*, *we managed by borrowing money… but now people like us have to survive with an insufficient amount of food during this lockdown*‥ *Families like us who rely on daily wages may look like rich from the outside but in reality*, *even we do not have enough to feed our family*. *Many families like us have to cut down their meal size by half compared to our food consumption in normal times… I have heard that those with bigger families had to skip meals due to lack of food because they have no land for agriculture*, *no job*, *and no alternative sources of income*.

                 *(Participant 1*, *47 years*, *Female*, *Saptari district)*

*Almost all households in our community are facing food scarcity and therefore we have to cut on our meals*. *People in our community do not have agricultural land or farm*. *We depend on daily based wages… we do not have any alternative source of income*. *We have already taken loans from our landlords*, *received food packages but now we have no idea where to seek help as it has been such a long time for this virus… It was very difficult to manage food for their family*. *If this situation continues like this then we may die without food*.

                 *(Participant 39*, *50 years*, *Male*, *Mahottari district)*

### Food relief and emergency support during the COVID-19 pandemic

Food relief and emergency support from the national government and Non-governmental organizations (NGOs) have played an important role in supporting people from low socioeconomic conditions in the community. Nepal’s government has pledged millions of Nepalese rupees to provide food relief to the most vulnerable in society. However, lack of consensus between the local parties has affected the distribution of the relief packages. A ward chairperson explained this as:

*Budget from the provincial government had been provided and both budgets from provincial and local government were used for food relief packages for needy households*. *According to the provincial rule*, *there needs to be a consensus between the political parties at the local level for distribution of the food reliefs*. *We had to identify 250–300 most vulnerable people in the community*, *but the local parties didn’t come to a common understanding to prepare the list and therefore there were difficulties in the distribution of the food relief package*. *As a result*, *people in the community didn’t receive food relief packages due to disputes among different political parties in the local community*.

                 *(Participant* 4, *48 years*, *Male*, *Saptari district)*

These sentiments were echoed by the local people in the community. Many participants in this research highlighted the decision to distribute food relief packages only to the most vulnerable population in the community has led to conflicts in relief distributions. They asserted that other people, who were also affected by the ongoing pandemic crisis and were struggling to meet the food security needs of their families, expected themselves to be included in the distribution list for food relief packages.

*Food relief was not distributed in our community because of conflict*. *palika (local authority) prepared the list and only poor people were included but other families also had a problem and they have opposed the process of food distribution*.

                 *(Participant* 2, *21 years*, *Female*, *Saptari district)*

People who benefited from the relief packages were also not happy with the support, as the pandemic continues and the food relief they had received was not enough to last longer, especially for people from the disadvantaged community with bigger family size.

*Yes*, *a food relief package was distributed to poor families which included 25 kg rice*, *2 kg pulse*, *2-liter oil*, *and 2 packets of salt*. *With bigger families*, *this food was enough only for 10 days and after that people had to manage on their own*.

                 *(Participant 8*, *43 years*, *Female*, *Saptari district)*

Moreover, participants complained that not all the families that were in need received adequate support through the relief package.

*We received food relief 2 times*, *however*, *it was not enough*. *Local authorities told us that they do not have enough foodstuffs to distribute again and again as it is uncertain when this lockdown or virus will end*. *Also*, *the food relief packages were not distributed to all needy households and there was bias in the distribution of the relief packages*.

                 *(Participant 14*, *45 years*, *Female*, *Siraha district)*

Many participants highlighted that food reliefs distributed within the community were not enough and only people with political affiliation and contacts benefitted from these food relief packages.

*We have 900 households*, *but only 50% of households got the relief package and many households were still waiting to receive the relief*. *The local leader said we have not voted for him so we will not get relief*. *Their close ones easily received relief packages*. *We are very much frustrated with this discriminatory activity in food distribution*.

                 *(Participant 33*, *47 Years*, *Male*, *Rautahat district)*

*Only those were benefited who have direct contact with political party leaders*. *Not all poor people were not benefited*. *The food Relief package was totally biased*, *and the political party has driven*.

                 *(Participant 18*, *32 years*, *Male*, *Sarlahi district)*

The lack of inclusiveness while distributing food relief packages angered many people in the community and in some cases, this has resulted in the looting of those relief packages.

*People were not satisfied with the food relief packages distribution*. *it was distributed very late and was not enough for all the people in need* … *About 400 households had food scarcity but only 200 packages were provided from our local authority*. *People were very angry and those who did not receive the food relief packages looted them when the local municipality was transporting the food to distribute to the community*.

                 *(Participant 10*, *24 years*, *Female*, *Parsa district)*

Some participants highlighted that the relief packages have included poor quality foodstuffs. In many cases, this has not been detected but when identified, it has created further health risk for the poor and vulnerable families in the community.

*Our municipality office had bought poor quality food relief materials to distribute in our community*. *The district quality control office examined the foodstuffs and declared that all those relief food packages were spoiled and inedible*. *As soon as the local authority got the result*, *they wanted to get rid of these foodstuffs to avoid any further investigation and damage political credibility*. *Overnight*, *the local municipality office took all those several tons of foodstuffs to the nearby riverbank and they dumped them inside the ground*. *When local people came to know about this*, *poor families dug out all those dumped foodstuffs and brought them home to consume it and to avoid the severe food insecurity they are suffering from*. *When asked*, *they said that the quality control officers have tested only the sample from a few sacks and they have not tested all sacks*. *This looks good and feeds us so we are digging it out*.

                 *(Participant 36*, *51 Years*, *Male*, *Bara district)*

### Impact of COVID-19 and food insecurity on health and wellbeing

Most participants in this research felt anxious about the COVID-19 due to the loss of income, which has directly affected their physical or mental health situations.

*People are worried about the virus*‥ *they have lost jobs due to the lockdown*‥ *Expenses have increased and there is no alternative income source*‥ *we are facing an economic crisis*. *Mental problems are increasing in every household*‥ *Wages-based labourers like us are very depressed now*‥ *farmers and poor families are vulnerable to increasing mental health problems*.

                 *(Participant 32*, *40 Years*, *Male*, *Siraha district)*

Many participants in this research highlighted that the loss of daily wages due to COVID-19 has increased the frustrations, anxiety, and depression among many males, as they have failed to provide food to their families. This has increased the issues of mental health and gender violence, especially among the poor and vulnerable families within the community. A Health worker describes this as:

*Many Dalits and migrant people who have returned from India due to the loss of jobs are struggling to manage the food requirements for their families*. *The lack of money and food has increased the mental health issues such as anxiety and depression among them*. *This has led to violence within their family*, *especially beating their wives*.

                 *(Participant 38*, *27 Years*, *Male*, *Mohattari district)*

Another health worker was concerned about the health status of these families, as there is a lack of nutritious food and in many cases lack enough food for the poor and vulnerable people in the community.

*The ongoing lockdown and lack of enough food relief packages from the local government have left the daily-wage earners and extremely poor people in a struggle to meet the daily food requirements for their families*. *The increase in food prices and the shortage of green vegetables and other food items have created further difficulties even for other families*. *Pregnant mothers are not getting enough food to eat*. *This pandemic has increased the risks of hunger and malnutrition and has severely affected the dietary intake of all people in the community*.

                 *(Participant 34*, *27 Years*, *Male*, *Rautahat district)*

## Discussion

This study has explored food insecurity among people from the disadvantaged community and low-income families during the COVID-19 pandemic in Province-2 of Nepal. Using a social-ecological framework [27], the study has found that food insecurity during the COVID-19 pandemic was linked to various factors such as intrapersonal, interpersonal, organization, community, and public policy factors at different levels.

The intrapersonal factors include individual demographic characteristics such as ethnicity, socioeconomic variables, place of residence, and occupation of the participants, which were found to have an influence on food insecurity during the COVID-19 pandemic. Our study shows that families from poor socio-economic backgrounds and disadvantaged ethnic groups, who mostly relied on daily wage and low or unskilled labours, experienced increased food insecurity during the COVID-19 pandemic. Previous studies in Nepal conducted before the pandemic shows that Dalits, who are socially marginalized ethnic groups in the country, have been highly vulnerable to food insecurity as compared to other advantaged ethnic groups such as Brahmin and Chhetri [43–45]. In addition, a recently conducted study with civil society and the government authorities explicitly reflected that wages-based labourers, indigenous communities, and women from marginalized ethnic groups and geographically disadvantaged regions have suffered severe forms of food insecurity and malnutrition during the COVID-19 pandemic [24]. This may be due to the reason that Dalits in Nepal have poor access to material and non-material resources because of financial constraints, which might have put them at higher risk of food insecurity [46]. The majority of Dalit families are landless and depend mostly on daily wages for their survival forcing them to be trapped in the intergenerational poverty cycle [46]. In past, different levels of governmental and non-governmental programs were directed to the welfare of Dalit ethnic groups in Nepal. However, such programs most often had been poorly funded or inadequately implemented [47], which has been exposed and exacerbated by the COVID-19 pandemic. This has been reflected by the participants within our study, where they shared their experiences of how the pandemic has affected their family economic status, food-deficit, and health and wellbeing.

The interpersonal factors such as weak family support system, lack of friends or peer support, limited food storage at home, and food habits during the COVID-19 pandemic were found to be linked with food scarcity among the participants in our study. This may be due to the increasing number of migrant workers returning to Nepal during the COVID-19 pandemic, which has affected the flow of remittance making the left behind family members vulnerable to the economic needs that are necessary for the food security of the families [20]. In this study, those families whose major source of income was foreign remittance were found to have experienced hunger and food insecurity during the COVID-19 pandemic. The rapid increase of remittance in preceding years has suddenly dropped during pandemic resulting in poor income status of the family left behind in Nepal [48]. This result is also similar to the most recent survey report by the government of Nepal that revealed about one-fourth of migrant worker’s families in Nepal were found to have inadequate food consumption during the COVID-19 pandemic [16]. This situation could be explained by the evidence that nearly one in every three households in Nepal relies on foreign remittance for their livelihoods [17]. The lockdown imposed in most of the countries across the world during the COVID-19 pandemic has either resulted in the loss of migrant worker’s jobs or delayed their payments that have directly impacted on food security of their family members staying back in their homeland [20].

The organizational factors such as limited availability of safe and nutritious food, market closures, transportation disruption, raised food prices in local stores, and challenges to the local food production were some of the factors that were found to have influenced food insecurity among disadvantaged and low-income families in the province 2 of Nepal. The current study discovered that food prices during the lockdown period were unexpectedly increased that reduced the purchasing capacity of poor households further compelling such families to cut down the size of their regular family food requirements. The recent United Nation report illustrated that food prices in the cities areas around the world were suddenly increased as disruptions occurred in the food supplies especially from rural farming areas due to imposing lockdown during the pandemic [49]. Also, the findings from the high-income countries showed that about one-third of the total food insecure households during the COVID-19 pandemic were due to a sudden rise in food prices and a poor support system for vulnerable families [50]. This might have happened due to the unprecedented suspension of all types of work when restriction mobility was imposed to control the COVID-19 transmission. As a result, poor families relying on regular income for their livelihood started to face immediate penury that directly impacted their food security status. The economic shock due to the pandemic among poor families may have long-term multiple adverse consequences in countries like Nepal where about one-fifth of households are poor and one in every two were food-insecure even before the COVID-19 pandemic [25, 31]. The findings from the study conducted in India also support our results where their study revealed that the COVID-19 pandemic has expected to push a significant number of the population below the poverty line that may have negative consequences on their livelihoods and health in the future [51]. The evidence from a previous study conducted in Bangladesh shows that extreme poverty was rare before the COVID-19 pandemic; however, the pandemic and subsequent lockdown has seen the income of about half of families fall below $1·90 per day [52]. This is associated with a concerning reduction in food security and an increase in moderate and severe food insecurity [52].

The lockdown imposed within and outside Nepal has caused the closure of borders and disrupted the cross-border transportation services severely affecting the supply of food commodities [16]. Nepal is highly dependent on India for importing foods. In the midst of the crisis, “Indian producers halted rice exports to Nepal in early April [53]. This desperate situation might play a significant role behind food insecurity in a low-resource, high-agriculture country like Nepal. In addition, during the COVID-19 pandemic, farmers in province 2, and more widely in the Terai region of Nepal, were not able to get fertilizers on time and there was also a shortage of farm labours that have directly impacted the local food production [24]. The period between April and June is rice plantation season and most of the households during this period suffered from food shortages and relied on buying rice or staple foods from local mills [54]. Likewise, the previous study from Brazil also showed that ruptures in food production and disruption in the family agriculture food chain in the local environment exacerbated the household food insecurity during a pandemic [55].

The community factors such as weak social networks and support systems were seen to have disproportionately affected families from the low socioeconomic and disadvantaged community towards their food insecurity. The only social network for such families is to rely on their landlords who can provide and support their needs. In our study, most participants adopted different forms of coping strategies such as borrowing money from landlords, mortgages of assets, consumed less diversified and compromised food items and change their food behaviors during COVID-19 pandemic to meet their food requirements and differ with their occupations and socio-economic background. Similar results from the study conducted in Bangladesh reported that more than a fourth-fifth of households suffered from some levels of food insecurity during the lockdown and they adopted similar forms of coping strategies [56]. This is due to the reason that poor families in resource-poor countries basically rely on daily based causal labor work [57]. The majority of people in such countries are employed in informal sectors with limited provision of job security, social security, and insurance schemes [57]. In addition, low or middle-income countries economy was poorly protected and were less resilience to economic shock during the COVID-19 pandemic [58].

The public policy factors included support provided by the government at all levels. Most participants in this research reported that although the three-tier of the government of Nepal has released a huge amount of fund for the emergency food relief support to the needy population, poor consensus between local political parties has blocked the adequate distribution of the emergency relief affecting the people in need and exposing them to food scarcity. Bias in food relief distribution was widely felt by the community stakeholders in the region. Poor coordination between the newly formed three-tier governance structures, nepotism, and favoritism from local political parties have been the major reasons for unfair distributions of relief packages at local levels [59]. The report from National Disaster Risk Reduction Centre (NDRC) Nepal also showed the similar types of discriminations while food relief distributions during COVID-19 pandemic in another provinces of Nepal [60]. Likewise, similar discriminations in the identification of victims and distributions of emergency support were observed during the recent responses for earthquake-affected regions in Nepal [61]. The victims had visited several times local government authorities to release their support allocated under federal government policy [62]. Such discrimination and favoritism during relief distribution might have occurred due to widespread corruption, and poor accountability of local authorities [61, 62]. According to the Transparency International’s Corruption Perceptions Index (CPI), 2020, Nepal remained as one of the highly corrupt countries ranked at 117^th^ position [63]. However, few non-governmental stakeholders including civil society and local community-based organizations were found to have acted proactively in food relief package distributions to the most vulnerable groups of the population both in rural and urban settings. Moreover, despite the lockdown measure was imposed to control the transmission of COVID-19, there was lack of effective management to mitigate the adverse health and nutrition consequences among vulnerable population [39]. The previous study reflects, a long and strict lockdown in Nepal had negative consequences on population health and nutrition status [3].

All these five factors of the Social-Ecological Model acts at different levels but also interacts with each other to affect the health and wellbeing of the population. This study uncovered that different forms of mental health problems and gender violence were observed in the communities particularly among families who had poor resilience to economic shock during the COVID-19 pandemic. Our findings are comparable with similar results from a study from the neighbouring country of India where mental health problems, gender-based violence, and child abuse cases suddenly surged during the COVID-19 pandemic [64]. Likewise, the findings from the previous study conducted during the post-earthquake period in Nepal also showed a high prevalence of anxiety, depression, and suicidal ideation observed among vulnerable groups of the population [65]. Thus, the persistent mental health issues and poor wellbeing among vulnerable groups during COVID-19 might present long-term adverse mental health consequences [66]. This has highlighted the need for effective mental health services at the community level, as currently, Nepal’s healthcare system lacks adequate provision of mental health services [67].

## Strengths and limitations

The study captured the lived experiences related to the impacts of COVID-19 on food insecurity among low-income and disadvantaged families during the peak phase of the COVID-19 pandemic in one of the poorest Province of Nepal. The study included the opinions and experiences of diverse participants that provided useful evidence for immediate implications during and post COVID-19 pandemic crisis. However, the results of this study need to be interpreted in light of some limitations. First, this study captured the qualitative aspects of food insecurity in terms of community perception and experiences to accessibility, availability, and utilization of food, and therefore could not quantify the levels of food insecurity status. Second, the results present the context of food insecurity only during the COVID-19 pandemic period and may not represent the situation of normal time, though highlight the vulnerability of people from low socioeconomic and socially disadvantaged conditions towards their food security. As such many people have lost their jobs or stayed at home due to the lockdown and expressed their feeling of expectations for emergency support from concerned authorities.

## Conclusions

Food insecurity among low-income and disadvantaged families is a serious public health problem in the Province-2 of Nepal during the COVID-19 pandemic. The families who relied on daily based wages and remittance for their livelihood had mostly experienced food insecurity during the pandemic. Borrowing money and food from landlords, eating compromised or less diversified food, skipping the meals, and cutting the size of the meals were found most common coping strategies of food insecurity during the COVID-19 pandemic. Despite the government efforts in responding to the food crisis during the COVID-19 pandemic, essential support could not reach all vulnerable groups due to favoritism, nepotism, poor coordination between the three-tier of governments, and partiality done by local politicians and authorities in food relief distributions. Furthermore, participants also highlighted the risks of different types of nutritional and mental health issues among vulnerable population. Therefore, this study suggests the immediate identification of food insecure high risks groups and implementation of sustainable integrated food security strategies to prevent long-term hunger and malnutrition among vulnerable families in Province 2 of Nepal. Further, the practice of transparency, accountability, impartiality, and a good governance system is crucial for the effective delivery of emergency responses for food-insecure families during the COVID-19 pandemic.

## Supporting information

S1 Table Thematic network analysis framework (from codes to global themes). (DOCX)

## Abbreviations

COVID-19: Coronavirus Disease-2019
FAO: Food and Agriculture Organization
FCHVs: Female Community Health Volunteers
PCR: Polymerase Chain Reaction
PPE: Personal Protective Equipments
SMS: Short Message Services
USD: United States Dollar
USDA: United States Department of Agriculture
WFP: World Food Program
