# Supplementary material for: Food insecurity during COVID-19 pandemic: A genuine concern for people from disadvantaged community and low-income families in Province 2 of Nepal
Source: PLoS One. 2021 Jul 21;16(7):e0254954. doi: 10.1371/journal.pone.0254954 (PMC8294479; doi:10.1371/journal.pone.0254954)
Supplement: S1 Table — (DOCX) [file pone.0254954.s001.docx]

| **Codes** | **Basic themes identified** | **Organizing themes** | **Global themes** |
| --- | --- | --- | --- |
| - Anxiety - Fear - Pandemic - Transportation disruptions - Poor health - Food shortage - Affordability of food - Food prices - Local foods - Relief package - Community perceptions - Quarantine facility - Conflicts at quarantine - Mental health problems - Meat product - Ignorance - Isolation centers - Lack of resources - FCHVs support - management - Emergency plan - Favoritism - Anger and looting - Government response - Poor response - Sanitation and hygiene - Migrant returnees - Community expectations - Remittance - Transportation obstruction - Disadvantaged families - Job loss - Daily wages labours - Dietary diversity - Rice plantation season - Political disputes - Risk group - Maternal nutrition - Child nutrition - Coping strategies - Boarder closures - Fertilizers shortage - Staples foods - Family left behind - Community support - Local organization support - Government support | 1. Anxiety and fear among community 2. Community perception towards food insecurity 3. Maternal and child nutrition 4. Availability of foods in local markets 5. Challenges in food supplies 6. Disruption of food supply chain 7. Community perception towards food prices 8. Information on COVID-19 9. Risk towards eating poultry and meat product 10. Community support in relief distribution and supporting poor families 11. Closures of local food market 12. Roles of female community health volunteers in assessment 13. Impact among disadvantaged families 14. Availability of food 15. Jobs loss among daily based labours 16. Obstruction of transportation services 17. Female community health volunteers support during COVID-19 crisis 18. Government and non-government support during COVID-19 crisis 19. Open border crossing 20. Stop sending remittance to home country 21. Favoritism and political interference in food relief package management 22. Delay in food relief distributions 23. Community approach towards food insecurity coping strategies 24. Stakeholders involvement in planning and implementing food relief responses during COVID-19 | 1. Anxiety and fear among community due to food insecurity during COVID-19 pandemic 2. Disruptions of transportation services 3. Food shortage and unavailability of food in local markets 4. Increase in food prices in local shops and market 5. Government and non-governmental food relief support during pandemic 6. Female community health volunteers involvement in need assessment and food relief distribution 7. Community perceptions towards food relief package 8. Risk of deaths and malnutrition due to food shortage during COVID-19 pandemic 9. Poor monitoring at open border crossing between India and Nepal 10. Poor quarantine facilities and other food supplies 11. Delayed in food relief distributions 12. Discriminatory behaviors from local authorities in relief distributions 13. Poor mental health and well-being among disadvantaged families 14. Impact of remittance on families left behind | 1. Impact of COVID-19 on Food Security 2. Food insecurity and coping strategies during the COVID-19 pandemic 3. Food relief and emergency support during the COVID-19 pandemic 4. Impact of COVID-19 and food insecurity on Health and Wellbeing |

**Supplementary file S1: Thematic network analysis framework (from codes to global themes)**
